# Deformation Instabilities and Lamellae Fragmentation during Deformation of Cross-linked Polyethylene

**DOI:** 10.3390/polym11121954

**Published:** 2019-11-28

**Authors:** Zbigniew Bartczak, Alina Vozniak

**Affiliations:** Centre of Molecular and Macromolecular Studies, Polish Academy of Sciences, Sienkiewicza 112, 90-363 Łódź, Poland; awozniak@cbmm.lodz.pl

**Keywords:** deformation, semicrystalline polymer, polyethylene, crosslinking, deformation mechanisms, buckling, lamellae fragmentation

## Abstract

The effect of the topology of the amorphous phase and phase interconnectivity on the stability of the deformation of semicrystalline polyethylene was investigated. The chain topology was modified by crosslinking the samples with electron beam irradiation. The samples were deformed by plane-strain compression, while the accompanying structural changes were monitored with X-ray and differential scanning calorimetry (DSC). At the true strain around of e = 0.3, the lamellar stacks parallel to the loading direction experienced microbuckling instability, which shortly led to the cooperative kinking of lamellae. Macroscopically, this showed up as the ‘second yield.’ Buckling is driven by the different stiffness levels of the hard and soft layers and their strong connectivity—for given layer thickness, the critical strain for buckling appeared proportional to the stiffness of the amorphous phase. Above e = 1.0, lamellae fragmentation was observed. This resulted from the localization of crystallographic slip, which was triggered by stress concentrations generated at lamellae faces by taut ‘stress transmitter’ (ST) chains. Accordingly, the fragmentation was found to be dependent on the surface fraction of STs at the amorphous-crystal interface: a low concentration of STs resulted in fewer but stronger stress concentrations, which led to earlier slip localization, followed quickly by lamellae fragmentation. The observed instabilities, either lamellae kinking or fragmentation, profoundly influenced the deformation process as well as the resultant structure. Both phenomena relieved much of the structural constraints imposed on deforming lamellae and make further strain accommodation easier.

## 1. Introduction

The outstanding mechanical performance of semicrystalline polymers, including their ability for large-scale plastic deformation, can be attributed to their unique morphology, consisting of crystalline and amorphous elements in the form of thin alternating layers. Additionally very important is the specific structure of layers—more or less regularly folded chains in crystalline lamellae vs. highly entangled chains constituting the amorphous phase—and the robust phase interconnectivity, which is provided by numerous chains intersecting the amorphous-crystalline interface and ensuring the extremely strong covalent bonding of adjacent amorphous and crystalline layers. These chains allow for the load transfer between neighboring lamellae across the stack. It is commonly accepted that the performance of semi-crystalline polymers crucially depends on such molecular links, which are called stress transmitters (STs) [1]. Therefore, detailed knowledge, not only about the individual phases but also the interplay between them, is essential for understanding the deformation habits of semicrystalline polymers.

The deformation of a semicrystalline polymer appears to be a complicated process, involving all elements of their complex morphology. In this process, different micromechanisms are activated at various stages [1,2,3,4,5]. It is well documented that the crystalline part primarily deforms through mechanisms of a crystallographic nature [2,4,5,6], including various modes of crystallographic slip (which is principally the dominant mechanism), twinning or stress-induced crystallographic (martensitic) transformation. These mechanisms generally appear similar to those operating in low-molecular crystalline solids. A strain-induced ‘melting-recrystallization’ has also been proposed as a plausible mechanism involved in deformation [5,7,8]. The plastic deformation of polymer lamellar crystals is accompanied by several mechanisms operating in the amorphous phase: interlamellar shear (slip), lamella separation, and rotations of the stacks of lamellae. These mechanisms support the deformation of crystalline lamellae. Moreover, interlamellar shear can substitute the lacking slip systems that would intersect the chain direction. The joint activity of all these mechanisms allows for a full accommodation of the strain [4]. The deformation mechanisms mentioned above can be employed in a complex deformation sequence, with particular mechanisms activated and terminated at specific strains. Furthermore, the active mechanisms can be adjusted with advancing strain due to interactions between the adjacent amorphous and crystalline layers, which are intimately connected and therefore forced to jointly deform. These interactions can also lead to some deformation instabilities, which in turn, may effect in opening new deformation paths and/or launch alternative mechanisms, previously inactive. Therefore, such instabilities may appear very important, sometimes essential, in the entire deformation sequence. Recent studies have pointed out a very substantial role of the amorphous phase, including the ST chains in the process of plastic deformation and a deep mutual relationship between the deformation of the amorphous and crystalline components [9,10,11,12,13,14,15,16,17,18,19,20,21,22,23,24,25,26,27,28,29]. The role of the amorphous phase and its topology-dependent properties have turned out to be much more important in the deformation process than merely the role of a compliant medium transferring the load and adapting to the deformation of crystalline lamellae, as it was commonly assumed in the past. Amorphous phase layers have proven to be an active part of the structure, capable of tuning the deformation of crystalline lamellae. They can even take control of deformation, especially in the strain-hardening range, when the network stresses in amorphous layers become higher than the shear stresses needed to deform adjacent crystallites [11,13,19,30]. Frequently, the partial damage of crystalline lamellae has been observed in the same strain range—reaching the extensibility limit by the molecular network leads to a significant increase in resistance to the deformation of the amorphous phase and, consequently, to a substantial increase in stress [10,11,20,31]. The stretched ST chains, immobilized at the entry points to the crystal, give rise to localized stress concentrations on the lamellar faces. Because at this point the lamellae became notably thinner due to a well-advanced activity of the slip [2], these stress concentrations may trigger instabilities in that slip through its severe localization, which can quickly lead to lamellae disruption and its fragmentation into smaller blocks. Such fragmentation relieves much of the structural constraints imposed on deforming lamellae by their neighborhood and makes further strain accommodation easier. 

The main deformation instabilities found in the plastic deformation of semicrystalline polymers are associated primarily with cavitation, the formation of lamellar kinks, and lamellae fragmentation, which often lead to profound changes of the material morphology, as e.g., the transformation of the initial lamellar structure into microfibrillar due to lamellae fragmentation, frequently observed in tension. All of these instabilities are essentially related to the properties of the amorphous phase as well as to its stress response. For example, the lamella stacks respond to the tensile stress perpendicular to the lamella plane, initially with the dilatation of the amorphous layers, which can end up either with cavitation within this layer or with the cooperative bucking of layers, the formation of kinks, and, subsequently, the chevron-like morphology, depending on the properties of the amorphous layer, including its coupling to adjacent lamellae and additionally on the stress field, which can either promote or suppress internal cavitation [9].

While the cavitation, commonly observed in tension, has been studied (see, e.g., [32] and references therein), much less attention has been paid to other deformation instabilities, like microbuckling [29,33] or lamellae fragmentation [8,11,20,21,24,31]. This shortage motivated our recent study [33], in which we probed these phenomena with X-ray and differential scanning calorimetry(DSC) techniques and tried to find how the deformation process and its stability are influenced by the amorphous layers and its properties, including their connection to the adjacent crystalline layers. For this purpose, polyethylene samples of diverse structure and morphology were examined in plane-strain compression. Two main deformation instabilities accompanying plastic deformation were investigated: the microbuckling instability (observed around the true strain of e = 0.3–0.4) and the fragmentation of crystalline lamellae due to the localization of the advanced crystallographic slip (e = 0.6–1.0). Microbuckling leads to the cooperative folding and kinking of lamellae that were initially parallel to the compression direction; hence, this subsequently leads to their rapid and substantial reorientation. This transformation can show up macroscopically as the so-called ‘second yield.’ For a given stiffness of layers, which is practically constant within the studied set of samples, the microbuckling appears dependent on the ratio of the amorphous and crystalline layer thicknesses. The second instability—an extensive fragmentation of crystalline lamellae—has been postulated to be prompted by local stress concentrations generated at lamella faces by taut ST chains. The critical strain related to this phenomenon has been found dependent on the surface fraction of STs at the amorphous-crystals interface—when this fraction is low, fewer but stronger stress concentrations are expected, which apparently cause an earlier localization of the crystallographic slip [34] that cause the rapid local thinning of lamellae and quickly leads to their fragmentation. Both deformation instabilities deeply influence the further deformation path of the material and affect its final texture.

The goal of the present study was to extend the previous work [33] and investigate how modifying the topology in the amorphous phase can affect the course and stability of the process of plastic deformation of a polymer. In this previous work, the samples demonstrated various crystallinity and layer thickness levels but a similar topology of the amorphous phase, consisting of the network of entangled chains. In contrast, the polyethylene samples prepared for this study had a nearly identical crystalline phase and layers thickness but significantly differed in the chain topology within the amorphous phase. The macromolecular network in amorphous layers of samples used here consisted of both chain entanglements and chemical crosslinks, introduced by electron beam irradiation. As a consequence of solid chemical crosslinks, the amorphous component demonstrated various stiffness levels as well as the concentration of stress transmitters. 

All samples were deformed by compression in the plane-strain conditions using a channel-die, similarly to a process employed in the previous studies [18,19,20,31,33]. The main advantage of this deformation mode is its macroscopic homogeneity (no neck formation) and a lack of cavitation, which is prevented by the compressive stress components. Thus, the plane-compression allows for the study of the course of deformation that is not concealed by any other unwanted or inessential effects, in contrast to other deformation modes, e.g., tensile deformation, where the strong cavitation is usually observed. 

## 2. Experimental

### 2.1. Materials and Sample Preparation

The polymer used in this study was linear high-density polyethylene (HDPE) provided by Basell (Rotterdam, The Netherlands) of a weight- and number-average molecular weight *M_w_* = 170,000 and *M_n_* = 28,900, respectively, a density of 0.962 g/cm^3^, and a melt flow rate (MFR) = 0.2 g/10 min (2.16 kg, 190 °C). The oxidation induction time (OIT) of the resin (determined by means of DSC according to the ISO 11357-6:2018 standard) was much longer than 30 min, which proved the presence of stabilizers, added by the manufacturer to prevent the generation of unstable oxidation species or chain scission during processing. Therefore, no additional stabilization was needed. 

Samples in the form of 50 × 50 × 4.5 mm^3^ plates were compression molded at T = 190 °C and p = 50 bar. The molded plates were solidified by rapid cooling between two heavy aluminum blocks maintained at the temperature near 0 °C. All plates were produced according to the same protocol in order to obtain samples of similar structure and morphology. Part of the prepared plates was crosslinked by irradiation with an electron beam. Samples of various crosslink densities were obtained by irradiation with doses of 50, 100 or 200 kGy. The following procedure was used: The samples were packed in vacuum-sealed polyethylene bags and placed on a thermostated aluminum block, with one side exposed to an electron beam produced by the linear accelerator. Irradiation was performed by applying pulses of 6 MeV electrons at a frequency of 20 Hz (the duration of a single pulse: 4 ms). The average dose rate was 5.35 kGy/min, as determined by calorimetry. The required dose was obtained by adjusting the exposition time. In order to prevent an excessive heat generation within the polymer, the irradiation was carried out in steps up to 50 kGy each. Therefore, the irradiation of samples with 50 kGy dose was carried out in a single step, while the samples irradiated up to 100 and 200 kGy required two and four steps, respectively, with intermissions to cool down the samples. Other details are given in Reference [17].

Specimens of the desired size, suitable to compression experiments, were obtained by machining out of the neat and irradiated plates. The cutting tool and the machined material were continuously cooled with ethyl alcohol to prevent any unwanted modifications of its structure upon machining. Using this procedure, the outer near-surface layers, at least 0.25 mm thick, were machined out from each side of the plate. The core part of the plates, from which the specimens for compression tests were cut, demonstrated quite uniform spherulitic structure with no structural gradients [17,33,35]. The specimens prepared for channel-die compression had the dimensions of 50 × 10 × 3.85 mm^3^ (along the expected flow direction, the loading direction, and the constrained direction, respectively). 

### 2.2. Plane-Strain Compression

**Figure 1:**
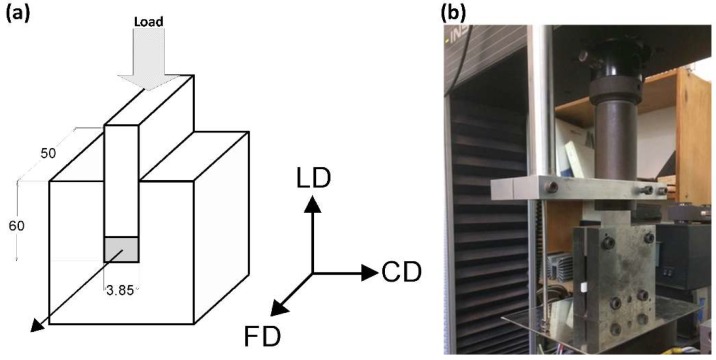
The channel-die fixture used for plane-strain compression: (**a**) schematic view—the compressed sample is marked gray. Dimensions are given in mm. The reference directions are the loading direction (LD), flow direction (FD) and the constrained direction (CD); (**b**) the photograph of the experimental setup. The part of the deformed sample (white) that has flown out of the channel is visible on the front.

Plane-strain compression was selected as the deformation mode for this study. Compression was performed by means of a computer-controlled universal tensile testing machine (Instron, Model 5582), equipped with the compression fixture of the deep channel-die type [18,31], as shown in Figure 1. The length of the channel was 50 mm, its width was equal to 3.85 mm, and the depth was 60 mm. The specimen filled the full length of the channel and fitted precisely its width. The specimen and die surfaces were lubricated to reduce friction during compression. The LVDT (Linear Variable Differential Transformer) displacement sensor was attached close to the specimen for precise strain determination. Other details are given in References [18,31]. 

Compression tests were performed at room temperature and a constant true strain rate of ė = 0.001 s^−1^, controlled by the software of the machine. The true strain (Hencky strain) was calculated from the measured load and plunger displacement data using the following equation:

(1a) $$e=\ln\left(\frac{h_{o}}{h}\right)=\ln\lambda$$

where *h_o_* denotes the initial height of the specimen, *h = h_o_* − Δ*h* is the actual height, Δ*h* is the measured displacement of the plunger, and *λ* *= h_o_/h* is the compression ratio. In the channel-die compression, the true stress is simply equal to the nominal stress since the area of the sample under load is always constant and equal to the plunger cross-section (the part of material gradually leaves the deformation zone in the channel due to plastic flow and then is no longer loaded). Therefore, the true stress was calculated with the formula:

(1b) σ=F/A

where *F* is the measured force and *A* is the surface area of the plunger.

The coordinate system used in this paper was related to the geometry of the channel-die: The loading direction (LD) is the direction of the compressive force applied to the plunger, the constraint direction (CD) is perpendicular to the sidewalls of the channel, and the flow direction (FD) is parallel to the channel length, i.e., directed towards its opening, as shown in Figure 1a.

### 2.3. Characterization

DSC (Differential Scanning Calorimetry): Thermal analysis was conducted using the TA 2920 DSC apparatus (TA Instruments, New Castle, DE, USA). The specimens, approximately 5–8 mg, were cut out from the middle part of the compressed specimen, perpendicular to the flow direction in order to minimize effects related to sample shrinkage. The melting thermograms were obtained at the heating rate of 10°/min. The weight crystallinity $X_{c}$ was calculated with the following equation:

(2) $$X_{c}=\frac{\Delta h_{f}}{\Delta h_{f100}}\cdot 100\%$$

where $\Delta h_{f}$ is the heat of melting of the sample determined from the melting thermogram and $\Delta h_{f100}$ = 293 J/g is the heat of melting of 100% crystalline PE [36]. The volume crystallinity $X_{v}$ was calculated using the equation:

(3) $$X_{v}=\frac{X_{c}\rho_{a}}{\rho_{c}-\frac{X_{c}}{100}\left(\rho_{c}-\rho_{a}\right)}$$

where $X_{c}$ is the weight crystallinity (wt.%) and $\rho_{c}$ = 1.0 g/cm^3^ and $\rho_{a}$ = 0.85 g/cm^3^ are the densities of the crystalline and amorphous phases, respectively.

WAXS (Wide Angle X-Ray Scattering): The effect of the plastic deformation on the average crystallite sizes was investigated using the four-axis X-ray diffractometer (DRON, Moscow, Russia) connected to a sealed-tube source (CuK_α_ radiation, *λ* = 0.154 nm), operating at 50 kV and 40 mA (Panalytical B.V., Almelo, The Netherlands). The θ–2θ scans were collected with the 2θ step of 0.05°. The average X-ray coherent crystal sizes were estimated from diffractograms determined for various specimen orientations with respect to the primary beam. They were calculated from the half-width of the respective diffraction peak using the Scherrer equation [37]:

(4) $$D_{hkl}=\frac{k\lambda }{\beta \cos\theta_{hkl}}$$

where $D_{hkl}$ is a crystallite size in the direction perpendicular to the (*hkl*) plane, *k* is constant (*k =* 0.94 when full width at half maximum (FWHM) is used as a measure of peak width, *k =* 1.05 for the integral width [37]), *λ* is X-ray wavelength, *β* is the half-width of a diffraction peak )corrected for instrumental broadening), and $\theta_{hkl}$ is the diffraction angle of the peak of interest. The peak separation procedure was applied to experimental diffractograms for accurate determination of the half-width of the peaks. The FITYK computer program, dedicated to peak-fitting [38,39], was used for this purpose. Crystalline diffraction peaks and amorphous halo were fitted with the Pearson VII and Lorentz functions, respectively. The (110), (200) and (020) peaks of the orthorhombic crystal form of PE (positioned at 2θ = 21.6°, 24° and 36.4°, respectively) were analyzed. Additionally, the (001), (200) and ($\bar{2}$01) peaks of the monoclinic form (2θ = 19.5°, 23.4° and 25.1°, respectively) were taken into account in calculations to obtain accurate fits. Other details are given in Reference [33]. 

SAXS: The lamellar structure of deformed samples was probed with two-dimensional small angle X-ray scattering (2-D SAXS). A camera with the sample-to-detector distance of 1.2 m was connected to an X-ray low divergence micro-source (CuK_α_; λ = 0.154 nm), operating at 50 kV and 1 mA, which was integrated with the multilayer collimation optics (GeniX Cu-LD by Xenocs SA, Grenoble, France; beam divergence of 0.8 × 0.8 mrad^2^). Two additional assemblies of hybrid scatterless slits (Xenocs) were placed between the collimation optics and the sample stage. The slits distanced 1.2 m each from the other formed the beam of the square cross-section, less than 1 mm^2^. The scattering of the sample was recorded with the solid-state area detector of the resolution of 172 × 172 μm^2^ (Pilatus 100K; Dectris, Baden-Daetwill, Switzerland). Other details are given in Reference [33]. The long period (LP) was determined from one-dimensional sections (background and Lorentz corrected) of 2-D patterns using Bragg’s law. The average thickness of the crystalline and amorphous layers was estimated from the LP and *X_v_* (calculated from DSC data) as *l_c_* = LP·*X_v_*/100% and *l_a_* = LP − *l_c_*, respectively.

## 3. Results

### 3.1. Structure and Morphology of Irradiation Crosslinked Samples 

The neat and crosslinked samples of the HDPE used in this work demonstrated the same uniform spherulitic morphology [17,35]. Table 1 shows the parameters of the crystalline phase in non-deformed samples, derived from DSC and SAXS data (the H-*n* sample code is used hereafter, where H stands for HDPE and *n* = 0, 50, 100 and 200 denotes the irradiation dose in kGy). Radiation with electrons is known to preferentially crosslink the amorphous phase, while the denser and ordered crystalline part remains practically free of chemical crosslinks [17]. Data shown in Table 1 confirm this view—the melting temperature of crystallites appeared practically independent on the absorbed dose. This also implies that the thickness of crystals is independent of irradiation, which was, in fact, confirmed by SAXS results—the long period, as well as the derived average lamellae thickness, remained practically the same in all samples. At the same time, only a small decrease in the degree of crystallinity with increasing irradiation dose could be noticed, which suggests the good stability of crystals with respect to the radiation treatment.

The properties of the macromolecular network in the amorphous phase were determined in the previous study [17] for the same set of HDPE samples. The concentration of crosslinks and the average molecular weight of the segment between crosslinks *M_c_* were evaluated there from the content of the insoluble gel and from the swelling data. The results of these estimations are reported in Table 2. Moreover, we previously demonstrated [17,18,35] that the molecular network of either entangled or crosslinked chains within amorphous layers can be characterized further on the basis of network stress. This stress can be determined from measurements of the residual stress still remaining in the deformed samples of a semicrystalline polymer, even after a very long relaxation period under constant sample height. That non-relaxing residual stress originates from the elasticity of the crystalline lamellar frame and the rubber-like network elasticity of amorphous layers. Consequently, measurements of the residual stress vs. applied strain allow for the estimation the effective network density *N_eff_*, which describes the volume concentration of STs of all types, which were active under applied conditions of deformation [35]. STs are the chains or chain sequences in the amorphous layer that connect the adjacent lamellae and are capable of carrying a load between them [1]. These include both tie-molecules that directly connect neighboring lamellae and other STs, constituting a sequence of several segments of different chains interconnected by chemical or physical crosslinks, e.g., chains immobilized with one end at crystal-amorphous interfaces of neighboring lamellae and mutually entangled or crosslinked at the other end. The above outlined approach, directly probing the network properties in amorphous layers, was applied to the studied samples of radiation-crosslinked polyethylene, and the obtained results were reported in the previous work [17]. Table 2 summarizes the obtained results of the effective network density *N_eff_* and derived average molecular mass between crosslink knots (either chemical or physical) *M_e_*, as well as the network modulus *G_n_*. 

The presented data show that the concentration of chemical crosslinks *N_x_* produced by irradiation linearly increased with increasing dose (this dependence correctly predicts *N_x_*≈ 0 for the dose = 0 kGy). The overall density of the network *N_eff_* appeared roughly equal to the sum of volume concentration of crosslinks *N_x_* and entanglements *N_e_* [17]: *N_eff_*≈ *N_x_ + N_e_*, whereas *N_e_* equals the density of the entangled-only network (the neat sample, H-0) *N_e_*= *N_eff_*(H-0). The value of *N_eff_*(H-0) = 3.9 × 10^26^ m^−3^ estimated for the neat sample H-0 is close to the entanglement density reported for polyethylene melt *N_e_*≈ 4.1 × 10^26^ m^−3^ (average molecular mass between crosslinks, *M_e_*= 1240) [40]. 

The effective network density *N_eff_* equals the volume concentration of STs active in amorphous layers. Therefore, the surface fraction of STs at the amorphous-crystal interface *F_s_*—defined as the fraction of the crystalline stems emerging from the crystal face that extend into the amorphous material of interlamellar layer as the stress transmitting chains—can be estimated using the following equation [35]:

(5) $$F_{s}=N_{eff}l_{a}\cdot s_{0}/2$$

where *s_o_*= 0.18 nm^2^ is the cross-sectional area of a single stem protruding from the crystal face. The calculated values of *F_s_* are listed in the last column of Table 2. It can be noticed that the concentration of stress transmitters significantly increases with increasing irradiation, from 0.14 in the neat PE to about 0.21 in that crosslinked with the 200 kGy dose.

### 3.2. Deformation Behavior

**Figure 2:**
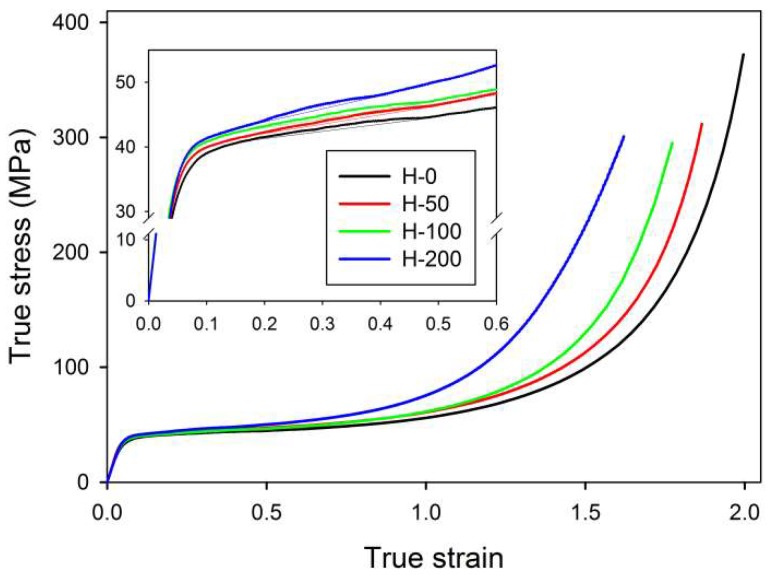
Representative true stress–true strain curves obtained in plane-strain compression with the constant true strain rate ė = 0.001s^−1^ at room temperature of the neat and irradiated polyethylene (PE) samples. The inset shows the enlarged initial part of the same curves.

The samples of virgin and crosslinked HDPE were deformed by plane-strain compression at a constant true strain rate of ė = 0.001 s^−1^ at room temperature. Figure 2 presents the obtained true stress—true strain curves, calculated from the measured load and displacement using Equations (1a) and (1b). These curves demonstrate the typical response for semi-crystalline polymers deformed in plane-strain compression. Both virgin and irradiated samples showed practically the same elastic modulus and a similar stress at yield. This similarity reflected their highly crystalline structure, which had practically not changed due to irradiation. Actually, the yield stress of the irradiated samples was slightly higher than of the neat sample. This may have reflected some contribution of the amorphous phase, which was notably stiffer in irradiated samples than in the neat material because of crosslinking (*cf*. network moduli reported in Table 2). In addition, a significant variation in the strain hardening behavior could be observed among the studied samples: the strain-hardening began at lower true strains, and, moreover, the true stress reached a very high level notably earlier (at lower strain) in the samples treated with an increasing dose, as could be expected for materials of higher network density [18,19]. As shown in our previous studies [18,31], the deformation of the crystalline component proceeds in a similar manner in all samples, involving the same crystallographic mechanisms, regardless of the crosslinking degree—it has been demonstrated that all characteristic changes in SAXS images resulting from important structure transformations occurred in neat and irradiated samples at the same strain level. These transformations included the cooperative kinking of lamellae, activated at a relatively low strain, below e = 0.4, which resulted in the formation of the four-point SAXS pattern, and the extensive fragmentation of lamellae, resulting in a new long period and the corresponding two-point pattern that develops along the FD in SAXS images at e > 1. Such deformation behavior agrees with the general deformation scheme suggested by Hiss et al. [11]. 

**Figure 3:**
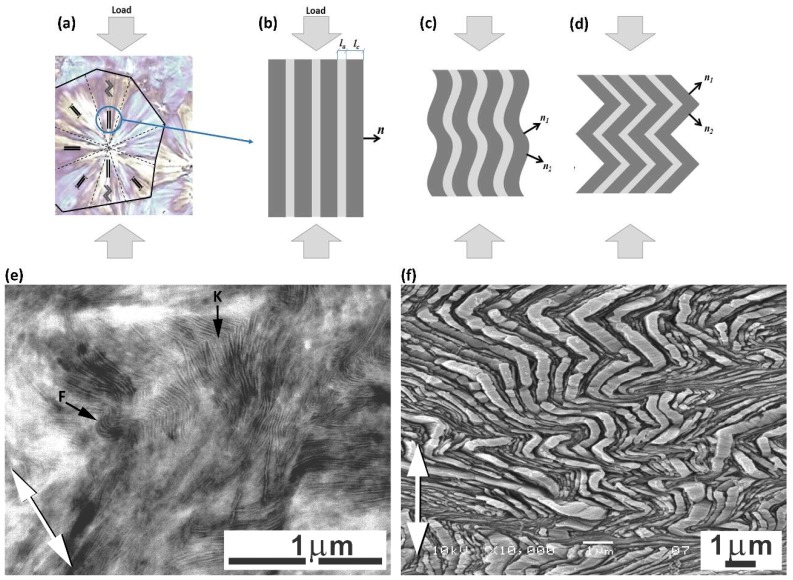
The schematic picture of the microbuckling mechanism (**a**–**d**) and exemplary electron micrographs illustrating the microbuckling and formation of kinks in polyethylene subjected to compression (**e**,**f**): (**a**) the location of stacks of lamellae parallel to compression direction that are susceptible to microbuckling and tilted lamella exposed to crystallographic slip—in the polar and diagonal sectors of a spherulite, respectively; (**b**) initial lamellar stack parallel to loading direction prior to microbuckling (n indicates the direction of lamella normal, which is also close to the chain direction in crystals, *l_a_* and *l_c_* denote the thickness of the amorphous and crystalline phases, respectively); (**c**) cooperative folds produced by microbuckling; (**d**) angular kinks that develop from folds. n_1_ and n_2_ illustrate the normal reorientation of lamellar n due to lamellae folding or kinking; (**e**) TEM micrograph of the high-density polyethylene (HDPE) sample compressed to e ≈ 0.8. The black arrow F points out the fold of lamellae, while K indicates the angular kink. The white arrow at the bottom of the micrograph indicates the compression direction (HDPE, *M_w_* = 120,000, similar to the HDPE used in this study, ultra-thin section stained with chlorosulfonic acid and uranyl acetate); (**f**) SEM micrograph of the same HDPE crystallized under high pressure of 480 MPa to obtain very thick lamellae and then compressed to e ≈ 1.0 (the surface etched with permanganic etchant prior to SEM observations).

Another feature observed in stress–strain curves is the so-called ‘second yield,’ which can be discerned in the form of a low and very broad local maximum extending from e ≈ 0.2 to e ≈ 0.45 and centered around e ≈ 0.3 in all samples—see enlarged curves, displayed in the inset of Figure 2. The second yield, similarly to the primary yield, is commonly associated with the deformation of the crystalline component. Initially, it was hypothesized that the second yield could be linked to the activation of the block slip acting along the chain direction (so-called ‘coarse’ chain slip), which can ultimately lead to lamellar fragmentation [41,42,43,44,45]. Sedighiamiri et al. [46] in turn, proposed to associate this second yield with transverse slip systems rather than with the coarse slip along the chain direction, on the basis of model calculations. In the recent study [33] we demonstrated that the second yield is most likely related to the buckling instability in stacked lamellae, which were initially oriented parallel to the load direction. Such microbuckling leads to the cooperative folding or angular kinking of these lamellae, resulting in the development of a chevron-like lamellar morphology in relatively small domains within polar sectors of deforming spherulites. Kinking, probably triggered by buckling instability, has been observed in many semicrystalline polymers, e.g., polyethylene (PE), isotactic polypropylene (iPP), polyoxymethylene (POM) or isotactic polystyrene (iPS) [47,48,49,50,51,52]. Figure 3 presents the scheme that illustrates the microbuckling mechanism and formation of lamellae folds or kinks, along with exemplary electron micrographs, which illustrate the operation of this mechanism in samples of polyethylene subjected to compression. 

The microbuckling and subsequent cooperative kinking of lamellae are very important for the further deformation behavior of a polymer, as they cause a swift reorientation of the involved lamellae and can cause the skeleton of stiff lamellae to collapse. These phenomena can prompt the initiation of new, previously inaccessible, deformation mechanisms in just reoriented lamellae—prior to kink formation, the lamellae parallel to the loading (compression) direction could not deform by crystallographic slip due to very low resolved shear stress (RSS) in the plane and direction of a potential slip system. However, their reorientation in kinks (see Figure 3c,d) led to an increase in the RSS and, consequently, to the initiation of the slip process, just like that already active in other lamellae, oriented obliquely to the LD (e.g., those in diagonal sectors of spherulites). This transition, releasing the activity of new mechanisms of plastic deformation, can macroscopically show up as the second yield point. 

### 3.3. Lamellar Structure

**Figure 4:**
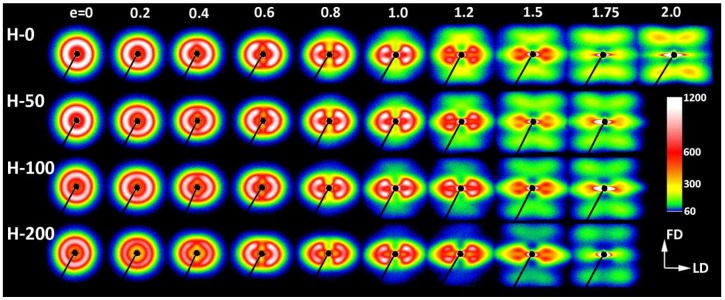
2-D small angle X-ray scattering (SAXS) images of the virgin (H-0) and irradiated samples (H-50, H-100, and H-200) deformed to the indicated true strain, obtained in the illumination along the CD. The color scale was selected to enhance the low-intensity features in patterns.

**Figure 5:**
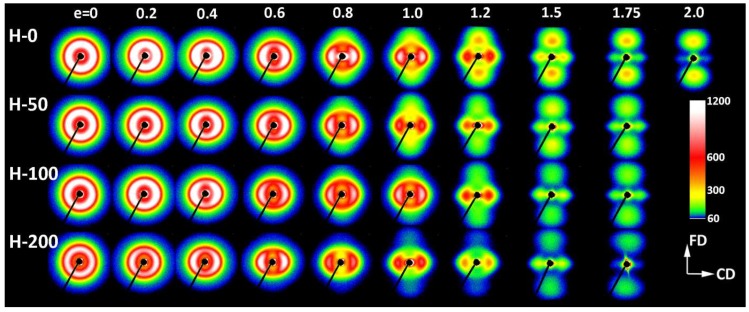
2-D SAXS images of the virgin (H-0) and irradiated samples (H-50, H-100, and H-200) deformed to the indicated true strain, obtained in the illumination along the LD. The color scale was selected to enhance the low-intensity features in patterns.

In order to better understand the deformation process and find characteristic changes in the structure related to deformation instabilities and the associated strains, samples with different crosslink densities were compressed to various true strains in the range from e = 0.2 to 2.0. The deformed samples were examined by means of X-ray and thermal techniques. In the beginning, the development of the lamellar structure with increasing strain was investigated with 2-D SAXS. Figure 4 and Figure 5 present the SAXS images obtained for the neat and irradiated samples, which were deformed to the true strains indicated in the plot. Two images were collected for each sample, with the X-ray beam illuminating the specimen either along the constraint direction (CD view images; Figure 4) or along the loading direction (LD view images; Figure 5). All images were recorded after sample unloading. 

**Figure 6:**
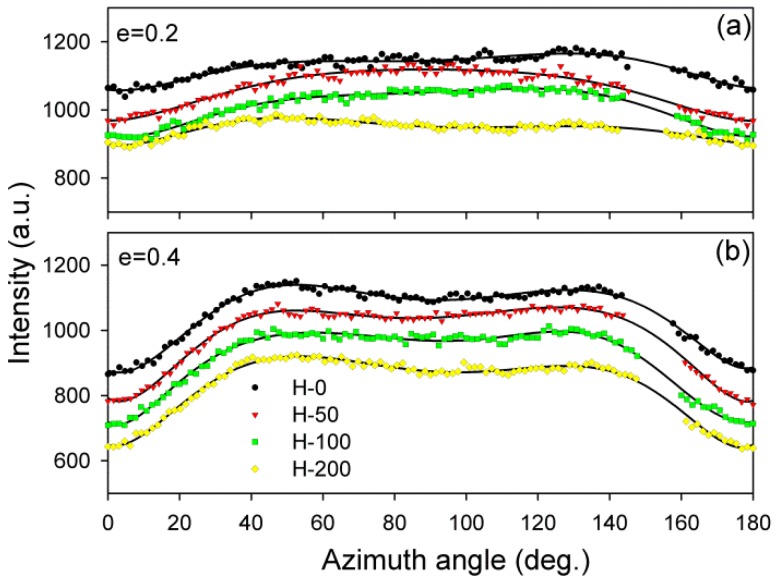
Azimuth scans of the SAXS images shown in Figure 4 (CD view) for samples deformed to: (**a**) e = 0.2 and (**b**) e = 0.4. The azimuth equals 0° for the FD and 90° for the LD. Curves were shifted vertically for clarity of presentation.

The images shown in Figure 4 and Figure 5 confirm that both the virgin and crosslinked samples deformed in a similar fashion, irrespective of the irradiation dose. The differences in their deformation paths seem rather subtle. The SAXS images of non-deformed samples exhibiting spherulitic morphology show uniform ring patterns, evidencing a lack of global orientation of lamellae. At a low strain, e = 0.2–0.4, SAXS images collected in both the LD and CD views transform into the elliptical form. The axis of the pattern parallel to the FD was slightly shortened compared to non-deformed samples, suggesting a small increase in the long period along the FD, probably due to dilation in the interlamellar amorphous layers that were oriented perpendicular to the FD, i.e., the mechanism of lamellar separation. Simultaneously, the scattering intensity observed along the FD began to decrease, which led to the transformation of the elliptical pattern into two separate arcs, which could already be discerned at e ≈ 0.2; see azimuth scans presented in Figure 6a. The rapidly decreasing scattering along the FD detected at e = 0.2–0.4 suggests a swift rotation of these lamellae that were initially normally oriented to the FD (i.e., lamellae parallel to the LD). In the range of e = 0.4–1.0, the broad arcs observed in the CD view images (Figure 4) progressively transformed into a four-point pattern—Figure 6b illustrates the formation of the maxima that clearly emerged along the elliptical envelope of the pattern, already below e = 0.4. These maxima were initially placed approximately 45–50° from the FD (e = 0.4) and rotated further away from the FD towards the LD, while increasing in intensity with strain increasing to e = 1.0. Concurrently, the arcs observed along the CD in the LD view images (Figure 5) narrowed significantly, which resulted in transformation of the LD view pattern into the two-point-type pattern, oriented along the CD. Above e = 1.0 the scattering intensity, including the intensity at maxima, began to gradually decrease, which could be observed in both the CD and LD views. This can suggest extensive damage of the lamellar ordering occurring in this range of strain. As is discussed later, such a fading of SAXS patterns was accompanied by a reduction in the average size of crystallites and overall crystallinity, detected by WAXS and DSC, respectively.

The four-point pattern, which appeared in CD view images above e = 0.4, suggests the development of two populations of lamellae, inclined symmetrically with respect to the FD and gradually rotating towards this direction with the increasing strain (i.e., lamellae normally rotating away from the FD). This four-point feature could still be distinguished in CD view images with increasing strain, even at the highest strain, e = 1.75–2.0, when the maxima rotate close to the LD and tended to merge and form a single equatorial streak along the LD. This indicates that a fraction of original lamellae, still undamaged, tended to orientate at a very sharp angle, nearly parallel to the FD, most likely due to highly advanced crystallographic slip along the chain direction. This slip caused the chain axis to rotate towards the flow direction, while lamella normally rotate in the opposite direction, towards the LD [2]. Concurrently, the two-point feature observed along the CD in the LD view images faded away and eventually almost disappeared at high strains (*cf.* Figure 5).

Another observation is that some new features emerge along the flow direction in SAXS images at the true strains exceeding 1.0: a two-line signature seen in the CD view images and a two-point feature, oriented along the FD seen in the LD view. These features indicate the development of a completely new component of lamellar ordering—the stacks of lamellae that are oriented perpendicular to the FD. Galeski et al. [20] suggested that this new orientation component is related to small blocks that are produced by the fragmentation of the highly deformed lamellae of the initial structure rather than to new crystallites formed in the strain-induced ‘melting-recrystallization’ process, postulated long ago by Flory [7]. The small blocks that survived the extensive lamellae fragmentation were presumably much less structurally constrained than the long lamellae of the initial structure and could therefore easily be rotated and undergo some restructuration to reduce the interface energy—the newly formed crystallites which had an excess of interface energy due to advanced deformation could now lower this energy purely by interface migration (without material rotation or shear) in a state of fixed molecular segment alignment. This process eventually led to development of the new long period along the FD. According to Galeski et al.: “Such restructuring should give rise to a new long period where the new topologically distorted (without lattice shear of the crystalline lamellae) lamellar fragments touch and associate” [20]. The new lamellae created in this way were significantly thinner than those constituting the initial structure (as indicated by the new long period, which was visibly shorter than the initial one), probably due to the large chain inclination that was produced by advanced chain slip in parent lamellae prior to their fragmentation and the formation of blocks. The scattering associated with the new long period was notably weaker than the scattering produced by the preceding lamellar structure. This indicates a relatively small number of these new lamellae that managed to arrange in stacks and give rise to the new maxima in the SAXS pattern. This population seemed to grow only slightly with increasing deformation at the expense of the progressive destruction of the preceding structure. 

**Figure 7:**
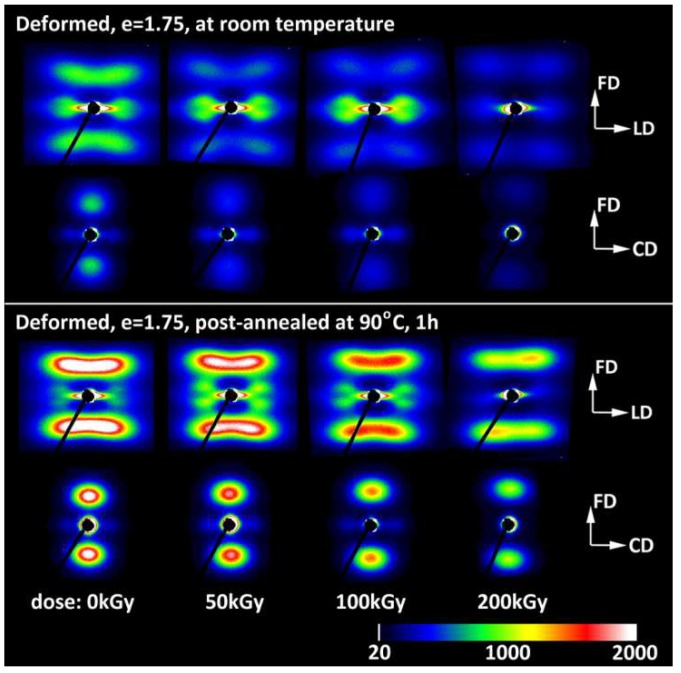
2-D SAXS images of the virgin and irradiated samples (dose indicated) deformed to the true strain of e = 1.75 at room temperature (top panel) and deformed at room temperature and post-annealed at T = 90 °C for 1 h (bottom panel), recorded in the illumination along the CD and the LD. The same intensity scale was used for all images.

The final lamellar structure observed at high strains in all samples studied (e = 1.75–2.0) could be pictured as the mixture of the original lamellae—now noticeably thinner due to to a well advanced slip mechanism, significantly fragmented, and arranged in a sharp chevron-like morphology aligned along the flow direction in the FD–LD plane—and newly developed stacks consisting of shorter and thinner lamellae. These new lamellae gave another contribution to the SAXS pattern, which suggested similar chevron-like lamellae orientation, although with a significantly larger apex angle. Nevertheless, both lamellae populations showed a similar preferred orientation of crystalline structure with the chain direction parallel to the flow direction [20,31] because the chains in ‘old’ lamellae were heavily tilted due to well-advanced chain slip [2,3,20], while this tilt could be significantly reduced in new blocks that had undergone restructuration [20]. Slightly different behaviors could be observed when the deformation was performed at a temperature higher than the α relaxation temperature. These conditions facilitate the heavier destruction of lamellae and the easier restructuration of the resulting blocks [31]. Consequently, the new ordering developed by these blocks nearly replaced the initial lamellar structure, like upon tension [20,31]. On the other hand, samples that were compressed at room temperature and then post-annealed demonstrated a significant (two-to-three fold) increase in the intensity of scattering produced by new stacks, yet the signature of the ‘old’ structure remained almost unchanged, as illustrated in Figure 7. This suggests either a more advanced restructuring of blocks that survived fragmentation or the crystallization of a brand-new species from highly oriented amorphous chains, both of which could have occured upon post-annealing, performed well above the α relaxation temperature.

**Figure 8:**
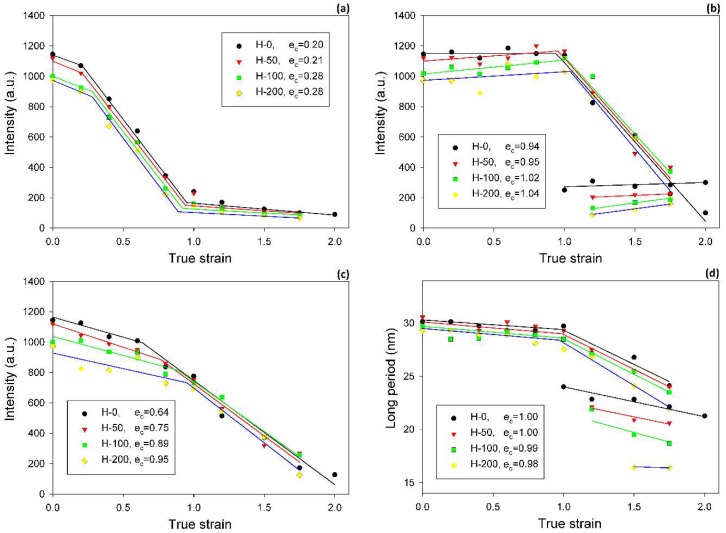
Strain dependencies of parameters determined from SAXS images (CD view) of HDPE samples irradiated with various doses, shown in Figure 4: (**a**) intensity observed along the FD in the main component of the pattern; (**b**) intensity at the maxima related to the main LP (solid line) and the new LP (dashed line) components; (**c**) the average intensity along the elliptical envelope of the main component; (**d**) the long period estimated from the position of maxima in the main and new LP components (solid and dashed lines, respectively). The straight segments represent the least squares fits calculated in the respective strain ranges. The critical strains determined as intersection points are reported in legends.

The inspection of SAXS patterns of samples deformed at room temperature, discussed above, suggested only minor differences in deformation habits of samples of various degrees of crosslinking. For deeper insight and finding possible differences, the CD view images were quantitatively analyzed. Each image was azimuthally scanned along an elliptical contour enveloping the dominant four-point pattern. From these data, the scattering intensity along the FD, the intensity at the maxima, and the average scattering intensity observed along the azimuth were determined. Because all samples demonstrated a very similar phase structure and the same thickness (set by the channel width in the channel-die fixture) and all measurements were carried out under the same beam conditions, it was possible to compare the respective intensities in the whole set of tested samples. In addition, the principal long period and the new long period developing at high strains were calculated from the 2θ angle corresponding to the respective maxima in the image. Figure 8 shows the parameters determined for samples of different crosslinking degrees, plotted as a function of the applied strain. In all graphs, the experimental relationships were approximated with straight line segments, which represented linear fits calculated in the respective strain ranges. The constructed plots demonstrate that all parameters considered here clearly changed with strain at certain characteristic points, indicated by intersection of the linear fits. These transition points observed for a given parameter were found at similar strain in all samples, regardless of the degree of crosslinking. The corresponding strains could be considered as the critical strains that indicated the activation of different deformation mechanisms and the associated transformations of the structure. 

The scattering intensity observed along the flow direction I(FD) in non-deformed samples was the same as in any other direction (uniform ring). As the strain increased, I(FD) initially decreased slightly, but above e = 0.2, it began to quickly descend, eventually reaching very low values below e = 1.0 (see Figure 8a). Then, at strains higher than 1.0, the plateau region of the low I(FD) was observed. Similar changes in I(FD) could also be observed in the LD view (*cf*. Figure 5). Because the intensity of scattering in a given direction is simply proportional to the population of lamellae of appropriate orientation, the reported observations indicated a quick vanishing of these lamellae, which were initially oriented with their normal along FD, starting at strain exceeding e = 0.2. This critical strain can be considered as the signature of the microbuckling and subsequent kinking of lamellae that led to a significant reorientation of the lamellae, which were initially oriented parallel to the LD (i.e., their normal alignment along the FD). The results showed that this critical strain for the initiation of microbuckling increased from e_c_(*I*_FD_) = 0.20 to e_c_(*I*_FD_) = 0.28 with the irradiation dose increasing from 0 to 200 kGy. 

Figure 8b illustrates a variation of the maximum intensity. Initially, this intensity gradually increased with strain in equatorial and diagonal areas of the SAXS image, and, then, at e ≥ 0.4, it continued to grow in the four maxima, which developed from equatorial arcs. The intensity at maxima increased up to the strain of e ≈ 1.0. This increase was linked with the preferred orientation of lamellae that developed as a result of the rotation of lamellae associated with active deformation mechanisms (primarily the crystallographic slip, supported by the interlamellar slip) [2]. The arising preferred orientation was revealed in a four-point feature that appeared in the CD view SAXS image. The maxima of the developed four-point pattern rotated towards the loading direction with increasing strain due to the activity of the slip mechanisms. However, at the true strain around e = 1.0, a breakdown in the intensity curve could be observed in all materials—the maximum intensity started to decrease sharply at this strain in every sample tested. The critical strain, indicated by the respective cross-over point, shifted to higher strains with the increasing irradiation of the sample, from e_c_(*I_max_*) = 0.94 to 0.95 and 1.02 up to e_c_(*I_max_*) = 1.04 for d = 0, 50, 100 and 200 kGy, respectively. This critical strain indicates the onset of the damage processes in the lamellae—note that the discussed reduction in intensity at the maxima of the four point pattern was not compensated by scattering in any other direction, which could be noticed in either the CD view or LD view images, where the intensity of scattering also seems to decrease. This observation is corroborated by examining the average intensity curves was determined along the main feature in the images in the CD view, which demonstrated similar cross-over point (*cf.* Figure 8c), although the associated strains (e_c_(*I_tot_*) ≈ 0.65–1.0) were lower than the respective critical strains derived from the *I_max_* vs. strain curves. The values of e_c_(*I_tot_*) were lower than e_c_(*I_max_*) and were probably related to an additional contribution to *I_tot_* of lamellae oriented in directions other than the preferred (which contribute to both *I_max_* and *I_tot_*). The population of such lamellae, including the lamellae initially oriented along the LD and which already disappeared (i.e., changing orientation) at low strains due to the formation of kinks, constantly decreased with strain.

### 3.4. Crystallite Size

**Figure 9:**
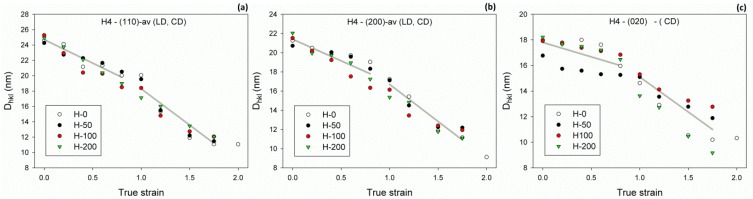
Dependence of the average X-ray coherent crystal size in the direction normal to the (110) plane (**a**); in the direction normal to the (200) plane (**b**); and in the direction normal to the (020) plane (**c**) on the applied true strain. The lines do not represent any real dependence and were drawn only to guide the eye.

On the basis of X-ray diffractograms determined for the deformed samples, the half-width of the main diffraction peaks—(110), (200) and (020)—was estimated, as described in the Experimental section. From half-width data, it could be seen that the average X-ray coherent crystal size was in the direction perpendicular to the (110), (200) or (020) crystal plane; this was calculated with the Scherrer equation (Equation 4). Because the samples deformed by compression exhibited a noticeable orientation of crystallites (texture), it was necessary to probe them with X-ray illumination along different directions—by specimen rotation or tilt—to more precisely estimate the average size of crystallites. In order to make this task simpler, it was decided to examine the specimens in only two orthogonal directions—along the LD and the CD, since it is known that plane-strain compression of PE results in crystal texture with single component—(100)[001], where most crystallites were preferentially oriented with their (*hk*0) planes perpendicular to the LD–CD plane: the normal to (200) plane oriented along the LD, the normal to (020) plane along the CD, and the normal to (110) plane in the LD–CD plane, near the CD [20]. It was assumed that probing the specimen along these two directions, followed by averaging the calculated parameters over the direction of probing, would provide an acceptable estimate of the average crystal size. Figure 9 shows the calculated average crystal sizes in function of the strain applied, determined for both the virgin and irradiated samples. It could be observed that the average sizes *D_hkl_* changed with strain in a similar fashion, regardless of the crystallographic plane taken into consideration—they tended to continuously decrease with strain, yet with the rate seen at high strains was notably higher than in the low strain range. The cross-over point of straight lines roughly approximating the experimental dependence could be located approximately at e_c_(*D_hkl_*) = 0.9–1.0 for all samples of various crosslinking degrees and crystallographic directions probed. The faster reduction of average crystal size above the critical strain e_c_(*D_hkl_*) could be interpreted as yet another signature of the lamellae fragmentation process that intensifies significantly above this critical strain. This coincides well with SAXS results reported earlier in this section as well as with previous reports on extensive lamellae fragmentation that was found to occur during the deformation of various polyethylenes in this range of the true strain [11,20,31,33].

### 3.5. Thermal Properties

**Figure 10:**
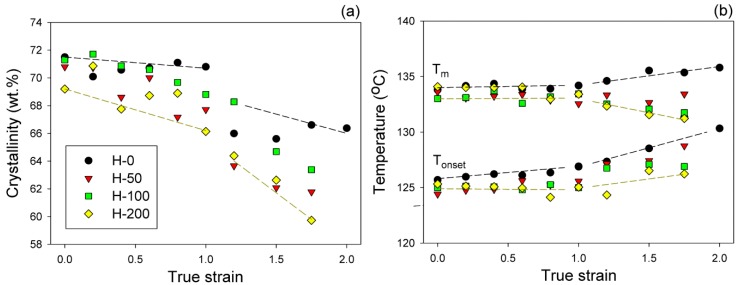
Dependencies of the crystallinity (**a**) and temperatures of onset and maximum of the melting peak (**b**) on the applied true strain determined for the HDPE samples crosslinked with an electron beam; the dose is indicated. Symbols represent experimental points, whereas lines were drawn only to guide the eye and do not represent any real dependence.

The melting behavior of raw and deformed samples was investigated by means of DSC. The crystallinity degree and the temperature of melting (onset—*T_ons_* and maximum of the melting peak—*T_m_*) were determined from the thermograms and then plotted in function of the applied strain; the respective plots are presented in Figure 10. For the severely deformed samples (e ≥ 1.75) a moderate decrease in crystallinity could be observed compared to non-deformed materials; see Figure 10a. However, the crystallinity of all samples remained nearly constant up to e ≈ 1.0, then suddenly decreased by about 2–4 wt.%, and next slowly decreased as the strain increased further. The strain, at which this sharp decrease began, can be regarded as a marker of partial damage of the crystalline phase (the critical strain e_c_(*X_c_*)), presumably again associated with an extensive fragmentation of lamellae. Figure 10b demonstrates that for all samples, the dependence of either *T_ons_* or *T_m_* on the applied strain could be approximated by two lines that intersected at e ≈ 1.0, irrespective of irradiation treatment. In the low strain range (e < 1.0) *T_m_* and *T_ons_* remained practically constant. However, above e = 1, the melting behavior diversified: *T_ons_* tended to increase with increasing strain in all samples, while *T_m_* varied with the strain depending on the irradiation dose—it tended to increase in the raw sample H-0, remained nearly constant in H-50, and tended to decrease in samples H-100 and H-200 as the strain increases. 

The reported changes in melting behavior and crystallinity due to deformation support the view of the damage of the lamellar structure induced by deformation through lamellae fragmentation. This fragmentation was a consequence of an advanced crystallographic slip, which tends to localize at some strain, preferentially in the thinnest parts of lamellae [20,31]. It involves the local destruction of lamellar crystals in their thinnest sections. Accordingly, this led to a sharp drop in *X_c_* and to an increase in both *T_ons_* and *T_m_*, as observed in the neat sample H-0. Extensive fragmentation seemed to start at e ≈ 1.0, along with partial destruction of the molecular network in the amorphous phase, which was also found to begin in this strain range in non-crosslinked material [18]. On the other hand, an increase of *T_ons_* seen together with a decrease in *T_m_*, as observed in irradiated samples, can suggest that along with damage of the thinnest lamellae (which begin to melt at *T_ons_*), a fraction of the medium and thick ones (which would melt near the maximum temperature *T_m_*) were also damaged in these crosslinked samples. Such behavior was interpreted previously [18] to be a result of the competition between two damage processes: crystals or network damage. Apparently, the destruction of crystals was easier than the damage of the robust network in amorphous layers of irradiated materials, consisting of chemically crosslinked chains, unlike in the neat sample, where the network erosion (partial destruction) by chain disentanglement appeared easier than heavy crystal damage [18]. 

The critical strains that can indicate the beginning of various deformation instabilities, estimated from the mechanical, SAXS, WAXS and DSC results reported in this section, are summarized in Table 3.

## 4. Discussion

In our recent paper [33] the deformation instabilities accompanying the plastic deformation of polyethylene of diverse structure and morphology were investigated. The first instability, observed at the true strain e = 0.3–0.4, was the microbuckling that led to the joint, cooperative folding or kinking of lamellae and frequently macroscopically manifested as the second yield. It was found that for a given stiffness of individual phases, the initiation of microbuckling depended on the ratio of the thickness of the crystalline and amorphous layers *l_a_/l_c_*, the dependence of which seemed to be consistent with the general concept that the buckling of layered materials is driven by different stiffness degrees of adjacent layers. At e = 0.6–1.0, another significant instability was observed: the fragmentation of crystalline lamellae due to the significant localization of the crystallographic slip. We postulated that the fragmentation was initiated in already highly deformed lamellae by the stress concentrations generated at the amorphous-crystal interface by stress transmitter chains, stretched and taut due to the interlamellar shear in amorphous layers that accompanies the deformation of crystalline lamellae. As a consequence, the critical strain for lamellae fragmentation appeared to depend on the surface fraction of stress transmitters at the interface (*F_S_*)—when *F*_S_ was low, the stress concentrations were fewer but grew stronger than in the system of high *F*_S_. This, in turn, could trigger an earlier localization of the slip, quickly leading to lamella break-up. Such instability had a large impact on further deformation of the material and strongly affected its final texture—the massive fragmentation of lamellae significantly reduced structural constraints imposed on deforming material and facilitated the formation of a new ordering of fragmented crystals along the FD.

In contrast to the previous work, where the samples demonstrated various degrees of crystallinity and layer thickness but had a similar topology of the amorphous phase, consisting of the network of entangled chains (although exhibiting different effective network densities) [33], the samples studied in this work exhibited a nearly identical crystalline phase (the same orthorhombic crystal modification, similar crystal thickness, degree of crystallinity, etc.) but significantly different topology in the amorphous phase, where the molecular network contained both chain entanglements and solid chemical crosslinks, introduced in various amounts by electron beam irradiation. As a consequence, the amorphous component in irradiated samples demonstrated larger stiffness and concentration degrees of stress transmitters, which were significantly higher than in the raw material (*cf.* Table 2).

The effects of buckling instability were observed in crosslinked samples in the range of low strain, similarly to the non-crosslinked samples studied previously [33]. This instability resulted in the development of the macroscopic second yield, which was observed in the true stress–true strain curves as a broad and low maximum, located at approximately e ≈ 0.2–0.45 (*cf.* Figure 2 and Table 3). In the same strain range, a steep decrease in intensity of scattering along the FD was detected in 2-D SAXS images (*cf*. Figure 4, Figure 5 and Figure 8a). This fast reduction of the scattering intensity along the FD indicates that the progressing deformation led to the rapid elimination of these lamellae that were oriented initially along the LD. Moreover, the four-point pattern developed in the CD view SAXS images at slightly higher strains could be easily observed at e ≥ 0.4 (Figure 4). These changes in SAXS images suggest a swift rotation of lamella initially oriented along the LD to the new preferred orientation, seen approximately 45–50° away from the FD. All observations reported above probably evidence the same mechanism, which, in our opinion, was the buckling instability, activated around e = 0.3 and quickly leading to the development of joint kinks in lamellae. The drop of SAXS intensity along the FD, which can be considered as the signature of initiation of such microbuckling and subsequent kinking, progressively shifted towards higher strain with increasing irradiation dose, from e_c_(*I*_FD_) = 0.2 (in H-0) to 0.28 (in H-200)—*cf*. Table 3. 

Buckling instability, resulting in the cooperative folding or kinking of layers, was observed in various layered materials in response to the compressive load along the direction of layers or to the tensile load, perpendicular to them. It seems to be a general phenomenon occurring in various materials on remarkably different length scales that can range from the molecular scale (e.g., liquid crystals) up to the macroscopic scale (like in geological formations) [9]. The precondition of buckling is layered morphology, which must consist of stacks of alternating soft and hard relatively thin layers. They must be also strongly coupled, especially when loaded in tension. In such systems, buckling originates from very different stiffness degrees of the soft and hard layers [9]. Buckling instability, ending up with the formation of lamellar kinks, has also been observed in semicrystalline polymers, e.g., in PE [47,48,49], iPP [29,50], POM [51] and iPS [52]. Angular kinks initiated by layer buckling cause only limited damage to the crystalline lamellae, mainly at the kink tip [33] (see also Figure 3f). Nevertheless, kinking appeared a vitally important phenomenon for the subsequent deformation behavior of the material—this induced a swift reorientation of the lamellae, which were not able to plastically deform earlier, and it additionally caused the skeleton of the stiff lamellae constituting initial structure to rapidly collapse. Prior to kink formation, the lamellae parallel to the compression direction could not deform by crystallographic slip due to very low resolved shear stress in the plane and the direction of any potential slip system. The reorientation of lamellae in kinks led to a significant increase in the resolved shear stress in the slip plane, which could initiate the crystallographic slip process in such lamellae, now becoming relatively easy, just like the crystallographic slip that was already been active in other lamellae that oriented obliquely to the LD, These phenomena opened up new paths for the plastic deformation of the whole material. Microbuckling and subsequent kinking, triggering new mechanisms of plastic deformation, often macroscopically manifest as the second yield point. 

**Figure 11:**
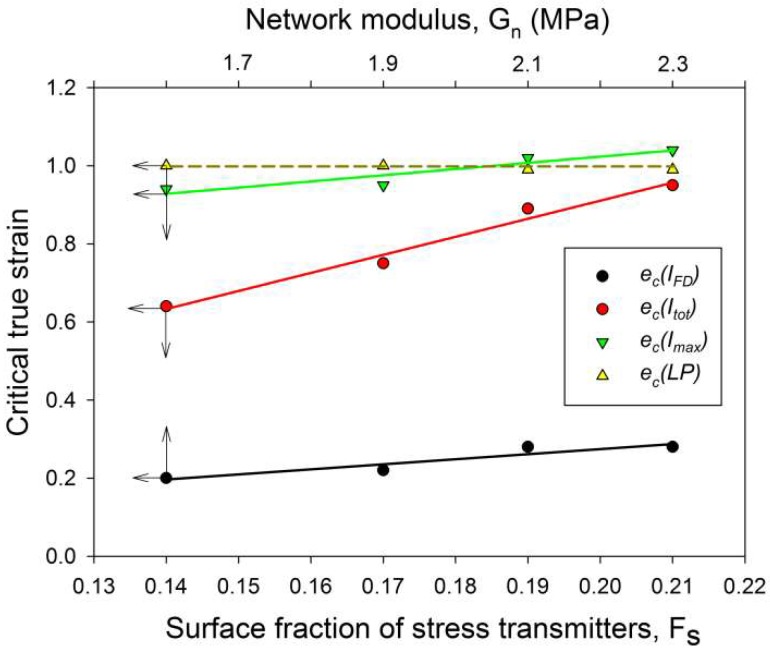
The dependence of critical strain for lamellae buckling on the network modulus of the amorphous phase e_c_(*I*_FD_) (●) and dependencies of critical strain for lamellae fragmentation on the ST fraction at the crystal-amorphous interface: e_c_(*I_tot_*) (*●*); e_c_(*I_max_*) (▼); e_c_(*I_LP_*) (▲), as derived from SAXS results. Arrows attached to the curves on the left side indicate their respective axes.

In a recent work [33], we found that for a given stiffness of the crystalline and amorphous phases, the initiation of lamellae microbuckling depended on the ratio of the amorphous and crystalline layer thickness *l_a_/l_c_*, and kinks started to develop at a lower strain when *l_a_/l_c_* decreased. In this study, the samples did not markedly differ in thickness of the amorphous and crystalline layers, as well as in degree of crystallinity, i.e., *l_a_/l_c_* and the stiffness of crystalline layers were roughly constant. Instead, they differed significantly in the properties of the amorphous phase, which exhibited increasing stiffness with increasing crosslink density. As reported above, initiation of microbuckling, indicated by the SAXS data, shifted toward higher strains with an increasing irradiation dose (increasing crosslink density). It can be concluded then that lamellae buckling was controlled in this case (*l_a_/l_c_* ≈ const, E_cr_ = const) by the stiffness of the amorphous phase, which increased with the dose—see Table 2, which reports the network modulus of raw and irradiated samples. The relationship between buckling instability and the stiffness of amorphous layers is illustrated in Figure 11, which presents the dependence of the critical strain e_c_(*I*_FD_) on the strain hardening (network) modulus of the amorphous component. The conclusion drawn here agrees with the general prediction that buckling is driven by different stiffness degrees of the layers (which are determined by the material stiffness of the respective phase and the layer thickness) [9]. If the stiffness of the crystalline and amorphous phase remains unchanged, then other morphological parameters, like the thickness ratio of hard and soft layers, can also play a role in the activation of buckling instability; this was indeed observed in the previous study [33]. 

Another deformation instability that was detected in crosslinked samples at higher strain, around e = 1.0, manifested as curve break points on the dependencies of the SAXS intensity (maximum or average), long period, average crystal size, crystallinity, and melting temperature on strain (see Figure 8, Figure 9 and Figure 10, respectively). All of these specific changes observed on the respective plots could be related to the same lamellae fragmentation phenomena, which were induced in this strain range by an advancing deformation [33]. Fragmentation results in partial damage to crystal ordering. Moreover, it can lead to the formation of new stacked lamellae that were thinner than the parent and oriented normally along the FD. 

One of the clear marks of lamellae fragmentation is a sudden decrease of crystallinity, here observed around e_c_(*X_c_*) ≈ 1.0 (Figure 10a). Crystallinity significantly fell in a narrow strain range, even by 4 wt.% in the neat sample H-0 and slightly less in irradiated samples. This means that the damage of a part of the lamellar crystals, most likely due to their fragmentation into smaller species, was accompanied by local, yet notable, destruction of the crystalline ordering. Such structure damage phenomena were reported earlier to occur in PE in this range of strain [11,20,31,33]. The increase of the onset temperature of melting, seen in the same range of strain (Figure 10b), suggests that fragmentation involved primarily the thinnest crystallites, which were apparently destroyed first. 

Another indication of lamellae damage by extensive fragmentation can be found in the relationship between the average crystal size and strain. The average X-ray coherent crystal sizes *D_hkl_* (estimated in various directions in crystal) tended to decrease with strain but with a varying rate, which appeared notably higher in the high strain range than in the low strain range (see Figure 9). The cross-over point could be located approximately at the strain of e_c_(*D_hkl_*) = 0.9–1.0, irrespective of the crystallographic direction. The faster reduction of average crystal size above this critical strain could be considered as another signature of lamellae fragmentation, which appeared to markedly intensify above e_c_(*D_hkl_*). This coincided well with the conclusion derived from DSC data, discussed earlier. Regrettably, any deeper analysis of the presented dependencies is problematic due to the noticeable scatter of data points. In particular, it is hard to assess whether the critical strain associated with a reduction of the average crystal size e_c_(*D_hkl_*) showed any dependence on the irradiation dose (hence, the density of crosslinks) or did not.

Some characteristic changes indicating lamellae fragmentation could be, furthermore, recognized in the lamellar structure at the strain around e = 1, when probed with 2-D SAXS. The CD view SAXS images of the deformed samples (Figure 4) demonstrated that the intensity in the maxima of the four-point pattern, which initially increased with increasing strain up to e ≈ 1, started to quickly decrease at higher strains, e > 1 (*cf*. Figure 8b). As reported in Table 3, the crossover point, determined from the plots presented in Figure 8b gradually moved to higher strain with increasing irradiation dose, from e_c_(*I_max_*) = 0.94 to e_c_(*I_max_*) = 1.04, for H-0 and H-200, respectively. The quickly decreasing intensity in the maxima of the pattern indicated a fast reduction of the population of lamellae stacks that were normally oriented in their current preferred orientation direction. This notion is supported by the results of the average intensity obtained by integration along the azimuth, which also showed a quick decrease of *I_tot_* at high strain, although for every dose, the critical strain e_c_(*I_tot_*) = 0.65–0.95 (Figure 8c) was smaller than the respective critical strain e_c_(*I_max_*) (*cf.* Figure 8b,c and Table 3). This was probably a result of the contribution of the scattering in directions around the FD, which already rapidly decreased at low strains because of microbuckling and kinking, as discussed earlier. 

Another cross-over point was found in the curve of the primary long period vs. applied true strain, approximately at e_c_(LP) ≈ 1.0—LP decreases notably faster at e > e_c_(LP) than below e_c_. The decrease of LP is associated with advancing plastic deformation by chain slip, which results in an increasing chain tilt in lamella and consequently decreasing lamella thickness, hence decreasing LP. The steeper decrease of LP above e_c_(LP) could have been a result of an extensive lamellae fragmentation, which seriously relieved the structural constraints that were imposed earlier on deforming lamellae by the initial structure. This makes the further deformation by slip mechanism in survived lamellae fragments easier and faster than in the initial, more constrained structure. Consequently, the thickness of these lamellae, and thus the LP, could decrease at a rate higher than prior to fragmentation.

The next consequence of lamellae fragmentation is the development of a completely new population of thin and short lamellae, preferentially oriented roughly perpendicular to the FD. They were formed by the restructuration of small crystalline blocks that survived the fragmentation of highly deformed initial lamellae [20]. The stacked new lamellae accounted for a new feature emerging in SAXS patterns right after lamella fragmentation, at e > 1.0. The scattering by these new elements of the crystalline structure had a look similar to the scattering produced by the fibrillar structure that usually completely replaced the initial lamellar structure upon drawing. However, the new lamellae formed in compression at room temperature did not completely substitute the initial structure and rather seemed to coexist with the remains of deformed and fragmented lamellae of the original structure, even at very high strains. The long period associated with the new lamellae was shorter than that associated with the initial structure, although it still seemed to depend on the original LP [33]. The scattering intensity of the new component of the deformed structure, once it appeared, increased only slightly with advancing deformation. This indicated that the short lamellae that contribute to this new structure component, probably formed in a single step rather than in a longer process, extended to a range of strains. As mentioned in the previous section, our observations demonstrate that the new lamellae arrangement arose from small blocks that survived the extensive lamellae fragmentation and from highly oriented amorphous chains in a process of some restructuring [20]. However, this process cannot be identified with often postulated ‘melting-recrystallization’ mechanism [7], because the thickness of the new lamellae seemed to depend on the thickness of the parent lamellae in initial structure rather than being related to the temperature of deformation as required in the strain-induced ‘melting-recrystallization’ mechanism [33]. 

The critical strains e_c_(*I_max_*) and e_c_(*I_tot_*) visibly increased with increasing irradiation. Regrettably, other critical strains related to lamellae fragmentation, derived from the SAXS, WAXS and DSC results, though located in the same strain range as e_c_(*I_tot_*), did not show such a clear dependence, perhaps due to the small variability and/or insufficient accuracy of estimates. Nevertheless, it seems reasonable to conclude just on the basis of the variation of e_c_(*I_max_*) and e_c_(*I_tot_*) that the instability of deformation, which brought extensive lamellae fragmentation, was correlated with the irradiation dose. On the other hand, that radiation dose determined the number of crosslinks, hence the properties of the amorphous phase, including the molecular network density *N_eff_* (consisting now of both chain entanglements and crosslinks) and the surface fraction of stress transmitters at the amorphous-crystal interface *F_s_*. In this way, a relationship was established between the deformation instability leading to lamellar fragmentation and the parameters describing the amorphous phase, either *N_eff_*, *F_s_* or *G_n_* (the network modulus). These parameters were discussed in the previous section and their values are reported in Table 2. The relation between *N_eff_*and *F_s_* is given by Equation (5). Since the thickness of the amorphous layer was practically constant in all samples studied here, the parameters *N_eff_*and *F_s_* became equivalent: *F_s_* = const·*N_eff_*. In addition, there was a linear dependence of *G_n_* on *N_eff_*: *G_n_* = kT·*N_eff_*. In Figure 11, which presents several critical strains derived from SAXS data and plots them in function of *F_s_*, a clear linear relationship between e_c_(*I_tot_*) or e_c_(*I_max_*) and *F_s_* can be observed. In the previous paper [33], we also concluded that lamellae fragmentation was controlled by the topology in adjacent layer, primarily through *F_s._* The following explanation was proposed: stress transmitter chains, when stretched out due to the shear in amorphous layer, which accompanies crystallographic slip, generate stress concentrations at the amorphous-crystal interface. Then, low *F_s_*result in fewer but stronger stress concentrations on the lamella face, which bring the localization of the crystallographic slip and a non-uniform ‘coarse’ deformation of the lamellae [34]. Consequently, localized slip quickly leads to lamella disruption and an earlier fragmentation of lamellae into small species. On the contrary, large *F_s_* result in smaller stress concentrations and thus a more homogeneous distribution of the stress on the lamella face, which promotes a more uniform, ‘homogeneous’ slip in crystalline lamellae and thus delays their fragmentation (due to other effects, e.g., non-uniform thickness) to a larger strain. The data presented in Figure 11 fully support the above findings.

## 5. Conclusions

The deformation study of linear polyethylene, crosslinked in the amorphous phase by electron beam irradiation and then highly deformed in compression, revealed several instabilities that accompany the plastic deformation process, occurring at various strains. These instabilities result in lamellae fragmentation and/or reorientation, which apparently enables the easier accommodation of the strain in an energy-minimizing way by opening new paths of relatively easy plastic deformation of the lamellar crystals. This prevents or delays excessive stress build-up due to high network stress generated in the amorphous phase and thus facilitates the further deformation of a polymer without its premature damage. The instabilities leading to lamellae fragmentation can have different origins and intensities that depend on the material structure—in both amorphous and crystalline phases and phase connectivity—as well as on the deformation conditions. 

One of the deformation instabilities studied in this paper is the microbuckling of lamellae and interlamellar amorphous layers that sets in at the true strain around 0.3 and results in their joint and cooperative bending that quickly leads to joint folds or kinks of lamellae. Kinking contributes to some fragmentation of lamellae, although limited to kink tips. What is important, however, is microbuckling followed by kinking induces a rapid and irreversible rotation of lamellae stacks, as well as the collapse of the stiff lamellar frame of the initial structure. This, in turn, allows for the activation and then the continuous operation of conventional mechanisms, like crystallographic slip, in these just reoriented lamellae. This transition frequently macroscopically manifests in the stress–strain curve in the form of low and broad local maximum, commonly reported as the second yield. Experimental evidence has shown that microbuckling is driven by a big difference in the stiffness of the soft (amorphous) and hard (crystalline) layers—in irradiated samples, in which the thickness of layers and elastic properties of the crystalline phase remain constant, the critical strain for buckling initiation depends linearly on the amorphous phase modulus. As found in the previous study [33], when the elastic properties of crystalline and amorphous components have not been altered, the strain at which the microbuckling was initiated depends on the ratio of the thickness of the amorphous and crystalline layers, though the thickness of the layer could vary. Such a relationship is consistent with the general concept that buckling in layered materials is driven by different stiffness degrees of adjacent layers (considered here as solid bodies)—the thick layer appeared stiffer than the thin layer of the same material, so the strain was related to buckling shifted to higher values. The current findings are in line with this conclusion. 

The second important instability of deformation studied here is the fragmentation of lamellae, initiated when the deformation of lamellae by crystallographic slip is already well advanced and stress concentrations develop at their faces due to stretched stress transmitter chains. This transformation occurs in polyethylene, also crosslinked, around a true strain of 1.0. The extensive lamellae fragmentation that relieves the deforming crystallites from constraints (at least partially) not only facilitates further deformation of survived lamellae or smaller crystallites but also can deeply transform (or even completely, as in the case of less constrained tensile deformation) the morphology of the material into a new ordering along the flow direction, as, e.g., the transformation of a lamellar morphology into a microfibrillar one, frequently observed upon tensile deformation. The lamellae fragmentation in crosslinked PE was found to depend on the surface fraction of stress transmitters at the amorphous-crystal interface *F_s_*, similarly to non-crosslinked samples studied previously [33]—fragmentation shifts to higher strains in materials of increasing *F_s_*, which is related to either increasing entanglement density or additional chemical crosslinking.

## Figures and Tables

**Table 1:** Structural characteristics of samples.

Sample, (dose)	Onset Temperature, *T_ons_* (DSC)	Melting Temperature, *T_m_* (DSC)	Weight Crystallinity, *X_c_* (DSC) ^(1)^	Volume Crystallinity, *X_v_* ^(2)^	Long Period, LP ^(3)^	Crystal Thickness, *l_c_* ^(4)^	Amorphous Thickness, *l_a_* ^(4)^
	(°C)	(°C)	(wt.%)	(vol.%)	(nm)	(nm)	(nm)
H-0 (0 kGy)	125.7	133.8	71.5	68.2	24.4	16.6	7.8
H-50 (50 kGy)	124.4	133.6	70.8	67.4	24.5	16.5	8.0
H-100 (100 kGy)	124.5	133.0	71.3	68.0	24.3	16.5	7.8
H-200 (200 kGy)	123.6	134.1	69.2	65.8	24.3	16.0	8.3

^(1)^ Calculated with Equation (2), Δ*H_f100_* = 293 J/g [36]; ^(2)^ Calculated with Equation (3); ^(3)^ LP determined with Bragg’s law from 1-D sections of 2-D SAXS patterns, background and Lorentz corrected; ^(4)^ The thickness of crystal and amorphous layers, *l_c_* and *l_a_*, calculated from the long period (LP) SAXS and the crystallinity *X_v_* (derived from DSC): *l_c_*= LP⋅*X_v_*/100%, *l_a_*= LP − *l_c_*.

**Table 2:** Parameters of the molecular network and derived from the network density.

Sample Code, (dose)	Chemical Crosslink Concentration, *N_x_* × 10^−26 (1)^	Effective Network Density in the Solid Sample, *N_eff_* × 10^−26 (2)^	Average Molecular Mass between Chemical Crosslinks, *M_c_* ^(1)^	Average Molecular Mass between Crosslinks, *M_e_*^(2)^	Network Modulus, *G_n_* ^(3)^	Fraction of STs at the Interface, F_s_^(4)^
	(m^−3^)	(m^−3^)	(g/mol)	(g/mol)	(MPa)	
H-0 (0 kGy)		3.9	-	1320	1.6	0.14
H-50 (50 kGy)	0.82	4.6	5940	1100	1.9	0.17
H-100(100 kGy)	1.28	5.2	3790	990	2.1	0.19
H-200 (200 kGy)	2.63	5.7	1840	910	2.3	0.21

^(1)^ Taken from the Reference [17], estimations based on swelling data; ^(2)^ Taken from the Reference [17], estimations based on fitting the residual stress-strain data; ^(3)^ The network modulus calculated as *G_n_ = N_eff_ kT*; ^(4)^ The surface fraction of stress transmitters at the crystal-amorphous interface, calculated with Equation (5).

**Table 3:** Critical strains estimated from the stress–strain curves (the second yield, e”), SAXS, WAXS and DSC data. The critical strains e_c_(*I*_FD_), e_c_(*I_tot_*), e_c_(*I_max_*) and e_c_(LP) were estimated as the cross-over points in the strain dependencies of the respective parameter: *I*_FD_—the scattering intensity along FD, *I_tot_*—the scattering intensity integrated azimuthally, *I_max_*—the maximum scattering intensity, and LP—the main long period, all determined from the CD-view SAXS images; e_c_(*D_hkl_*)—the critical strain found in the dependence of average X-ray coherent crystal size *D_hkl_*on strain (from WAXS); e_c_(*X_c_*), e_c_(*T_ons_*) and e_c_(*T_m_*)— the critical strains found in the strain dependencies of crystallinity, onset of melting, and melting peak temperature, respectively (from DSC melting thermograms).

Sample	e”-Range	Critical Strain								
		e”-max	e_c_(*I*_FD_)	e_c_(*I_tot_*)	e_c_(*I_max_*)	e_c_(LP)	e_c_(*D_hkl_*)	e_c_(*X_c_*)	e_c_(*T_ons_*)	e_c_(*T_m_*)
H-0	0.20–0.45	~0.36	0.20	0.64	0.94	1.00	~0.9–1	~1	~0.95	~0.9
H-50	0.20–0.45	~0.35	0.22	0.75	0.96	1.00	~0.9–1	~1	~1	~1
H-100	0.20–0.45	~0.35	0.28	0.89	1.02	0.99	~0.9–1	~1	~1	~1.05
H-200	0.20–0.40	~0.30	0.28	0.95	1.04	0.98	~0.9–1	~1	~1	~1
